# Self-efficacy and perceived recognition by peers, instructors, and teaching assistants in physics predict bioscience majors’ science identity

**DOI:** 10.1371/journal.pone.0273621

**Published:** 2022-09-22

**Authors:** Sonja Cwik, Chandralekha Singh

**Affiliations:** Department of Physics and Astronomy, University of Pittsburgh, Pittsburgh, PA, United States of America; PhD, PLOS, UNITED KINGDOM

## Abstract

Prior research shows that in a particular science domain, students’ identity depends on their self-efficacy, perceived recognition by others, and their interest in that domain. In this study, we investigated how the end of the semester physics self-efficacy and perceived recognition by others for bioscience majors enrolled in the second semester of a traditionally taught mandatory physics course sequence predict their overall science identity aligned with their disciplinary major. We find that bioscience majors’ physics self-efficacy and perceived recognition not only predict their physics identity but also their overall science identity. These relations between physics self-efficacy and perceived recognition and the overall science identity of bioscience majors suggest interdisciplinary connections that may provide additional pathways for boosting students’ science identity, e.g., by enhancing their self-efficacy and perceived recognition in their other mandatory courses such as physics. We also find that on average, women majoring in bioscience had lower physics self-efficacy, perceived recognition, physics identity, and overall science identity than men even though women were not underrepresented in the physics course. One possible reason is that the societal stereotypes and biases pertaining to who can excel in physics can impact women who are constantly exposed to them throughout their life.

## Introduction

Students’ science, technology, engineering, and mathematics (STEM) related motivational beliefs, e.g., self-efficacy and identity, have implications for their short and long-term outcomes in those disciplines regardless of their performance. Identity frameworks in STEM disciplines have been used to explore undergraduate students’ participation in college classes and career outcomes [1–8]. For example, in a study by Stets et.al., students with a higher science identity were more likely to enter a science occupation after college [9]. In addition, students on the pre-medical or pre-health track are more likely to have higher STEM identity and perceived recognition by others than their peers [10]. However, students on these tracks and bioscience majors, in general, are required to take physics courses for their major, and prior research suggests that it can be more challenging for women to form a physics identity than men [11–13]. While women are not underrepresented in these physics courses for bioscience majors, there may still be a gender gap in the motivational beliefs of students in the course. In a study on introductory bioscience courses, where women made up 60% of the enrollment of the courses, women participated less and underperformed on exams compared to the men in the courses [14]. Moreover, many bioscience majors take physics courses in their junior or senior year and the evolution in students’ motivational beliefs in the physics courses could also influence their overall science identity aligned with their major as well as future science pathways. Therefore, it is important to understand the connection between bioscience majors’ motivational beliefs in mandatory science courses, e.g., physics, and their overall science identity in order to contemplate multiple pathways to boost their science identity.

Science identity in this context refers to identifying with science or a student’s view about whether they see themselves as a “science person” [1, 15]. The science identity framework by Carlone and Johnson [2] includes three dimensions: competence (“I think I can”), performance (“I am able to do”), and recognition (“I am recognized by others”). Hazari et al. modified the framework specifically for physics. “Competence” and “performance” were defined as students’ beliefs in their ability to understand the subject and students’ belief in their ability to perform physics tasks. Additionally, recognition was framed as perceived recognition by others as students who can excel in physics. Lastly, a fourth dimension, interest, was added to the identity framework [16, 17]. Also, competence and performance were later combined into a single construct, self-efficacy or competency belief. In this framework, students’ physics identity was found to be influenced by their self-efficacy, interest, and perceived recognition [12, 13, 18–20].

Self-efficacy (closely related to competency belief) is defined as a person’s motivational belief that they can succeed in a particular activity or course [21, 22]. It has been shown to impact students’ engagement, learning, and persistence in science courses [23–28]. In particular, students with high self-efficacy are more likely to enroll in more challenging classes because they view difficult problems as challenges they can overcome as opposed to threats to be avoided [21]. However, in introductory physics courses in which women are underrepresented, studies have found a gender gap in self-efficacy favoring men that widens by the end of the course [29, 30].

Another motivational belief hypothesized to influence identity in a particular discipline, interest, may also affect students’ perseverance, persistence, and achievement [31–36]. One study showed that changing the curriculum to stimulate the interest of the female students helped improve all students’ understanding at the end of the year [37]. Within expectancy-value theory, interest and competency beliefs are connected constructs that predict students’ academic outcomes and career expectations [38].

The third factor influencing identity, perceived recognition by others, has been shown to be an important factor in motivation [39]. In one study, students’ perception of teacher recognition and support was more strongly linked to the motivation and engagement of girls than boys [39]. Studies have shown that female students are not recognized appropriately even before they enter college [11, 19, 40]. For example, one of the stereotypical views is that science is for high achievers or naturally gifted students [11], and in general, being a genius or exceptionally smart is attributed to boys [41]. Boys and girls are exposed to these fixed intelligence views starting from an early age [40]. These views impact recognition by others. One study found that science faculty members in biological and physical sciences exhibit biases against female students by rating male students significantly more competent even when the accomplishments of the hypothetical female and male students were identical [42]. Thus the science identity of women in college science courses can be negatively impacted by their lower perceived recognition by others [43].

All three of these factors (self-efficacy, interest, and perceived recognition) play an important role in identity formation. However, identity can be context dependent [3] and thus a student’s physics identity is not the same as their overall science identity aligned more with their disciplinary major. For example, bioscience majors may answer survey questions about their science identity based on their identity aligned primarily with their disciplinary major while physics identity is likely to be aligned with their views of physics and associated courses. However, since physics is also a science domain, it is possible that a positive or negative change in students’ physics identity in a course will also impact students’ science identity. For example, in one study, first-year college students’ math and physics identities were important predictors of their engineering critical agency and identity [13]. Since physics self-efficacy, perceived recognition by others, and interest that contribute to physics identity could also impact students’ overall science identity aligned with students’ disciplinary major, these connections are important to investigate as they may provide additional pathways for boosting students’ overall science identity.

**Fig 1:**
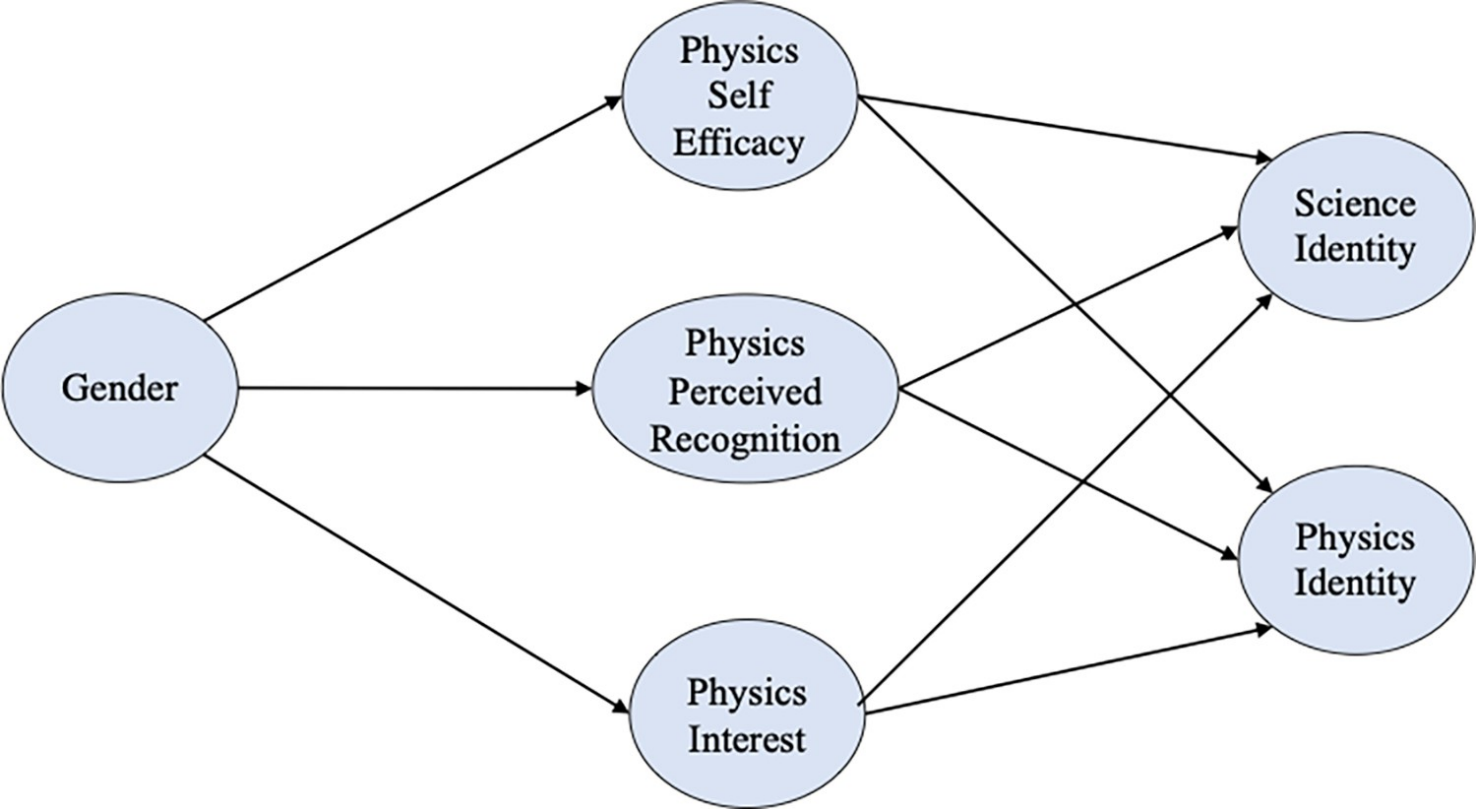
Schematic representation of the path analysis part of the model based on the theoretical framework. From left to right, all possible paths were considered. The regression paths from gender to physics identity or science identity are not shown since we did not find them to be statistically significant in our analysis.

Here we use the framework in which self-efficacy, interest, and perceived recognition play a central role in identity formation. Fig 1 shows a schematic representation of the path analysis part of the model in this study. In particular, we administered a motivational beliefs survey to students at the end of an algebra-based physics course sequence for bioscience majors and used mediation analysis in structural equation modeling (SEM) to investigate how physics self-efficacy, interest, and perceived recognition predict physics identity as well as the overall science identity aligned more with students’ disciplinary major. In this study, we answer the following research questions.

## Research questions

**RQ1** Are there gender differences in students’ motivational beliefs (physics self-efficacy, interest, perceived recognition, and identity, and overall science identity)?
**RQ2** To what extent can the components of student physics identity (i.e., self-efficacy, perceived recognition, and interest) predict students’ overall science identity?
**RQ3** What is the covariance between the physics identity and overall science identity?

## Methods

### Participants

The study was conducted at a large, public research university. We analyzed the results from responses to motivational beliefs survey administered to 873 students who took the survey at the end of the semester in an introductory algebra-based physics 2 course over two years. This research was carried out at the University of Pittsburgh in accordance with the principles outlined in the institutional review board (IRB) ethical policy, which approved the study. All interviewed participants provided oral consent for this research, which was documented orally and which was approved by the IRB. This class is taken by bioscience majors for whom both physics courses in this sequence are mandatory and is usually taken in their junior or senior year of undergraduate studies. Both physics courses in this sequence were traditionally taught with lectures as the primary mode of instruction with a few clicker questions per week. The university provided demographic information such as age, gender, and ethnic/racial information using an honest broker process by which the research team received the information without knowledge of the identities of the participants. The gender data provided by the university includes only binary options of “male” and “female” for students. We recognize that gender is a socio-cultural and nonbinary construct; however, we are limited to the binary data provided by the university in this study. Based on the university data, 38% of the participants identified as male and 62% as female students.

### Instrument validity

The motivational belief survey instrument used in this study is a validated instrument. This study focused on students’ physics self-efficacy, interest, perceived recognition, and identity as well as their overall science identity for the students enrolled in the physics 2 course using the validated survey. The *physics self-efficacy* questions measured students’ confidence in their ability to solve and understand physics problems [44–47]. The *interest in physics* questions measured students’ enthusiasm and curiosity to learn physics and ideas related to physics [46]. The interest questions were taken from activation labs fascination survey and the Deputy Director, Susan Gregory, gave permission to republish the questions. The *perceived recognition* questions measured the extent to which the students thought that other people see them as a physics person [44]. The *science identity* question evaluated whether the students see themselves as a science person. The *physics identity* questions evaluated whether the students see themselves as a physics person [44]. The physics and science identity instruments only included one question, which is consistent with past studies since it has been difficult to make other questions that factor in this category in exploratory factor analysis [1, 13, 48, 49]. The questions in the study were designed on a Likert scale of 1 (low endorsement) to 4 (high endorsement) [50].

The survey was adapted from previous research [45, 51] and questions were re-validated in our own context using Exploratory Factor Analysis (EFA), Confirmatory Factor Analysis (CFA), one-on-one student interviews [52], and Pearson Correlations. In order to test the reliability of the survey, we calculated Cronbach alpha [53]. The survey questions for each construct and factor loadings (lambda) for each question are given in Table 1. During one-on-one interviews, bioscience majors noted that their overall science identity was mostly aligned with their disciplinary major.

**Table 1:** Survey questions for each of the motivational constructs along with factor loadings (lambda) from the Confirmatory Factor Analysis (CFA) result for all students (N = 873). The rating scale for most of the self-efficacy and interest questions was NO! no yes YES! while the rating scale for the physics identity and perceived recognition questions was strongly disagree, disagree, agree, strongly agree. All *p*-values (showing statistical significance for each factor loading) are < 0.001.

Construct and Item	Lambda
Science Identity	
I see myself as a scientist.	1.000
Physics Identity	
I see myself as a physics person.	1.000
Physics Self-Efficacy	
I am able to help my classmates with physics in the laboratory or recitation.	0.610
I understand concepts I have studied in physics.	0.704
If I study, I will do well on a physics test.	0.742
If I encounter a setback in a physics exam, I can overcome it.	0.686
Physics Interest	
I wonder about how physics works. ^††^	0.693
In general, I find physics. ^†^	0.793
I want to know everything I can about physics.	0.778
I am curious about recent discoveries in physics.	0.723
Physics Perceived Recognition	
My family sees me as a physics person.	0.914
My friends see me as a physics person.	0.910
My physics instructor and/or TA sees me as a physics person.	0.684

^†^ The rating scale for this question was very boring, boring, interesting, very interesting.

^††^ The rating scale for this question was never, once a month, once a week, every day.

The pair-wise Pearson correlations are given in Table 2. Pearson’s *r* values signify the strength of the relationship between variables. The inter-correlations vary in the strength of their correlation, but none of the correlations are so high that the constructs cannot be separately examined. The highest intercorrelation was the value between Physics Identity and Perceived Recognition (0.82). Perceived recognition questions ask about external identity (perception of whether other people recognize an individual as a physics person), whereas the physics identity question asks about internal identity (whether an individual sees oneself as a physics person) so past research has found a high correlation between these constructs [1, 13, 20, 54]. However, the correlation is low enough that they can be considered separate constructs [55]. Furthermore, the correlations between the physics-specific constructs (perceived recognition, self-efficacy, interest, and physics identity) are stronger than the correlations between them and science identity. In addition, Cronbach alpha was used to measure the internal consistency of the items. The Cronbach alpha is 0.78 for the self-efficacy questions, 0.83 for interest questions, and 0.87 for perceived recognition questions which are considered reasonable [53].

**Table 2:** Pearson inter-correlations are given between all constructs based upon student responses to the motivational beliefs survey at the end of physics 2.

Pearson Correlation Coefficient				
Observed Variable	1	2	3	4
1. Perceived Recognition	--	--	--	--
2. Self-Efficacy	0.57	--	--	--
3. Interest	0.57	0.58	--	--
4. Physics Identity	0.82	0.58	0.56	--
4. Science Identity	0.23	0.26	0.24	0.21

### Analysis

We first compared female and male students’ mean scores for all predictors (self-efficacy, perceived recognition, and interest) and outcomes (physics identity and overall science identity) for statistical significance using *t-*tests and for the effect size using Cohen’s *d* [56]. Cohen’s *d* is *d* = (*μ_m_*−*μ_f_*)/*σ_pooled_*, where *μ_m_* is the average score of male students, *μ_f_* is the average score of female students, and *σ_pooled_* is the pooled standard deviation for all students. To quantify the statistical significance and relative strength of our framework’s links (see Fig 1), we used Structural Equation Modeling (SEM) as a statistical tool by using R (lavaan package) [57]. The SEM is an extension of the multiple regression, which affords conducting several multiple regressions simultaneously between variables in one estimation model (with the possibility of multiple outcomes, e.g., both physics and science identities). This is an improvement over multiple regression since it allows us to calculate the overall goodness of fit and allows for all estimates to be standardized simultaneously so there can be a direct comparison between different structural components. We report model fit for SEM by using the Comparative Fit Index (CFI), Tucker-Lewis Index (TLI), Root Mean Square Error of Approximation (RMSEA), and Standardized Root Mean Square Residuals (SRMS). Commonly used thresholds for the goodness of fit are as follows: CFI and TLI > 0.90, and SRMR and RMSEA < 0.08 [58].

The model estimates were performed using moderation analysis to check whether any of the relations between variables show differences across gender by using “lavaan” to conduct multi-group SEM [59] Initially we tested different levels of measurement invariance in the multi-group SEM model with gender moderation. In each step, we fixed different elements of the model to equality across gender and compared the results to the previous step using the Likelihood Ratio Test [59]. Since we did not find significant moderation by gender, we tested the theoretical model in mediation analysis, using gender as a variable directly predicting all constructs (physics self-efficacy, perceived recognition, interest and identity, and overall science identity) to examine the resulting structural paths between constructs.

## Results and discussion

In relation to **RQ1**, women had statistically significantly lower mean values than men for all constructs in our model, including science identity (Table 3). Both women and men scored lowest in physics identity and women scored in the negative range (i.e., a score of 2 which corresponds to “disagree”). One hypothesis for the gender gap in these constructs (indicated by their lower scores in Table 3) is that women may be affected by previous experiences, stereotypes, and biases about who belongs in physics and science in general and who can excel in it, which can accumulate over their lifetime. Table 3 shows that the gender gaps remain at the end of physics 2. It is also clear from Table 3 that both female and male students’ science identity is on average higher than their physics identity. This is reasonable since interviews with some of the students suggest that bioscience majors’ overall science identity is closely tied to their disciplinary major and they saw themselves as scientists primarily because of their interest in bioscience.

**Table 3:** Mean construct values by gender as well as statistical significance (*p*-values) and effect sizes (Cohen’s d) by gender. No superscript means *p*-values are < 0.001 and superscript “a” means *p*-value = 0.006.

Predictors and Outcomes	Mean		Cohen’s *d*
	Male	Female	
Perceived Recognition	2.24	1.98	0.39
Self-Efficacy	2.94	2.73	0.40
Interest	2.77	2.31	0.73
Physics Identity	2.18	1.86	0.44
Science Identity	3.02	2.86	0.19^a^

After analyzing descriptive statistics, we used SEM to investigate the relationships between the constructs and to unpack whether the constructs contributed toward explaining student physics identity and science identity. We initially tested gender moderation between different constructs using multi-group SEM (between male and female students) to see if the relationships between the motivational beliefs were different across gender. There were no group differences at the level of weak and strong measurement invariance including no difference at the level of regression coefficients. Therefore, we proceeded to gender mediation analysis to understand how gender mediates physics identity at the end of the yearlong introductory physics sequence.

**Fig 2:**
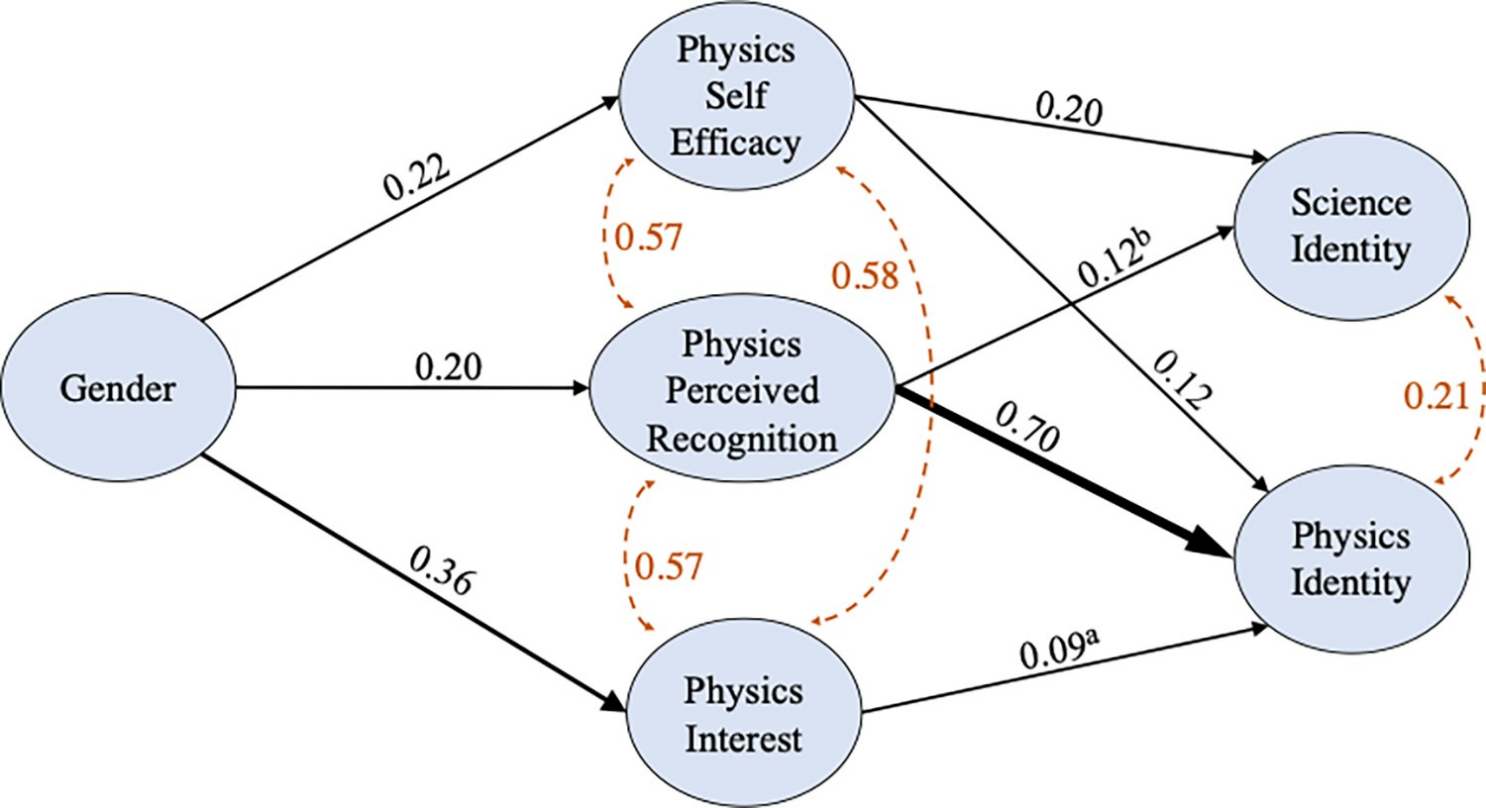
Result of the path analysis part of the SEM with mediation between gender and science/physics identity through physics perceived recognition (P.R.), self-efficacy, and interest. The line thickness qualitatively denotes the relative magnitude of the standardized regression coefficients β shown. The dashed lines indicate covariance. All *p-*values for β are indicated by no superscript for *p* < 0.001, “a” for *p* = 0.004, “b” and for *p* = 0.010.

The results of the SEM are presented visually in Fig 2. The model fit indices indicate a good fit to the data (acceptable fit thresholds in parentheses): CFI = 0.964 (> 0.90), TLI = 0.952 (> 0.90), RMSEA = 0.058 (<0.08), and SRMR = 0.038 (< 0.08). The solid lines represent regression paths and the regression coefficients (β) represent the strength of the regression relation. All three of the intervening variables (perceived recognition, self-efficacy, and interest) predict physics identity at the end of the yearlong physics course sequence, similar to past models [1, 54]. Perceived recognition has the largest direct effect on physics identity with smaller effects from self-efficacy and interest. In response to **RQ2,** we find that students’ science identity is predicted by physics self-efficacy and perceived recognition with student self-efficacy in physics having the largest direct effect on science identity (0.20). For bioscience majors, this physics 2 course is the second of the two physics courses they take, usually in their junior or senior year. In addition, gender is directly connected to perceived recognition (P.R.), self-efficacy, and interest. The relations between gender and both science identity and physics identity are mediated by the intervening variables and gender does not directly predict either science identity or physics identity. Thus, women have a lower physics and science identity because they have lower self-efficacy, perceived recognition, and interest. In response to **RQ3,** we investigated the covariance (dashed lines) between science and physics identity and find that science identity and physics identity covary with one another (0.21). Thus, while students’ overall science identity and physics identity are not the same construct, they could influence one another in meaningful ways.

## Summary and implications

In physics courses that bioscience majors are required to take for their major, women have lower mean values in their motivational beliefs than men (Table 3). It is important to note that there was a high correlation between students’ physics perceived recognition and physics identity (Table 2), however, that is consistent with past research [1, 13, 20, 54]. In addition, the science and physics identity constructs included one question which is consistent with past studies since it has been challenging to make other questions that factor in this category in exploratory factor analysis [1, 13, 48, 49].

In our model, we found that some of the motivational beliefs that predict physics identity also predict science identity at the end of the course. The identity model in Fig 2 shows the relationship between the predictors (self-efficacy, perceived recognition, and interest) and students’ physics and science identities. In particular, we find that at the end of a two-semester mandatory physics course sequence, bioscience majors’ physics self-efficacy and perceived recognition not only predicted their physics identity but also their overall science identity primarily aligned with their disciplinary major. The model suggests that physics TAs and instructors can potentially play an important role in increasing not only students’ physics identity but also their overall science identity, since perceived recognition by them in the physics course predicts science identity. It is important to note that physics self-efficacy also predicts science identity even though the students’ overall science identity is more closely related to their bioscience identity. These relations between physics self-efficacy and perceived recognition and the overall science identity of bioscience majors suggest interdisciplinary connections that may provide additional pathways for boosting students’ science identity aligned with their major, for example, by enhancing their self-efficacy and perceived recognition in their other mandatory science courses such as physics. Some ways instructors can build these interdisciplinary connections is by providing examples and problems in class that connect physics concepts with bioscience concepts. For example, in the introductory physics lab course, there are several labs about bioscience concepts including those focusing on the physics of human eyes, electrocardiogram (EKG or ECG), blood pressure, and viscosity measurements that help students build connections between physics and bioscience. Since science identity has the potential to influence students’ persistence in science careers, it is important for physics instructors to employ approaches that improve students’ science identity.

We also find covariance (0.21) between the overall science identity and physics identity which may be useful for devising strategies for boosting students’ science identity and career aspirations. Since physics 2 is usually taken in the students’ junior or senior year, it is one of the last non-major STEM courses they take before they graduate. Therefore, physics instructors and TAs may have the opportunity to improve students’ science identity by recognizing and affirming their students positively for making progress. Furthermore, at the end of a traditionally taught two-semester mandatory physics course sequence, we find that on average, women majoring in bioscience had lower physics self-efficacy, perceived recognition, physics identity, and overall science identity aligned with their disciplinary major than men even though women were not underrepresented in the physics course. One possible reason is that the societal stereotypes and biases pertaining to who can excel in physics can impact women who are exposed to these stereotypes and biases from an early age through differential upbringing, media coverage, K-12 education, and the culture of college physics classes. Moreover, although both men and women had an average perceived recognition below the positive lower threshold (score of 3 corresponding to “agree”), women’s averages were lower than men’s. The fact that women feel less recognized by their instructors and TAs than men could influence women’s self-efficacy, interest, and identity in physics in particular and science in general.

Physics instructors may unwittingly reinforce gender stereotypes about physics and communicate lower expectations for female students in physics classes. By not letting men dominate the conversations in class and explicitly praising effort and affirming women when they do well/achieve in the class, the gender gap in students’ perceived recognition could be decreased. This is especially important when implementing active learning pedagogy in the classroom. If not implemented using teaching strategies that are equitable and inclusive, the gender gap in the courses may increase [60]. In prior studies, men have been shown to dominate responding to questions in class and women have reported lower scientific self-efficacy [61]. Moreover, instructors should be careful not to say that problems are “trivial”, “easy” or “obvious” when students’ ask them for help after trying their best because otherwise female students are more likely to feel disparaged (or negatively recognized). Another way to improve the learning environment in science courses is through classroom interventions, which have been shown to eliminate the gender gap in performance and also have the potential to positively impact the motivational beliefs of students [62–65].
